# The binding specificity of Translocated in LipoSarcoma/FUsed in Sarcoma with lncRNA transcribed from the promoter region of cyclin D1

**DOI:** 10.1186/s13578-016-0068-8

**Published:** 2016-01-25

**Authors:** Ryoma Yoneda, Shiho Suzuki, Tsukasa Mashima, Keiko Kondo, Takashi Nagata, Masato Katahira, Riki Kurokawa

**Affiliations:** Division of Gene Structure and Function, Research Center for Genomic Medicine, Saitama Medical University, 1397-1 Yamane, Hidaka-shi, Saitama 350-1241 Japan; Institute of Advanced Energy, Kyoto University, Gokasho, Uji, Kyoto 611-0011 Japan; Graduate School of Energy Science, Kyoto University, Gokasho, Uji, Kyoto 611-0011 Japan

**Keywords:** TLS/FUS, Long noncoding RNA, pncRNA

## Abstract

**Background:**

Translocated in LipoSarcoma (TLS, also known as FUsed in Sarcoma) is an RNA/DNA binding protein whose mutation cause amyotrophic lateral sclerosis. In previous study, we demonstrated that TLS binds to long noncoding RNA, promoter-associated ncRNA-D (pncRNA-D), transcribed from the 5′ upstream region of cyclin D1 (CCND1), and inhibits the expression of CCND1.

**Results:**

In order to elucidate the binding specificity between TLS and pncRNA-D, we divided pncRNA-D into seven fragments and examined the binding with full-length TLS, TLS–RGG2–zinc finger–RGG3, and TLS–RGG3 by RNA pull down assay. As a result, TLS was able to bind to all the seven fragments, but the fragments containing reported recognition motifs (GGUG and GGU) tend to bind more solidly. The full-length TLS and TLS–RGG2–zinc finger–RGG3 showed a similar interaction with pncRNA-D, but the binding specificity of TLS–RGG3 was lower compared to the full-length TLS and TLS–RGG2–zinc finger–RGG3. Mutation in GGUG and GGU motifs dramatically decreased the binding, and unexpectedly, we could only detect weak interaction with the RNA sequence with stem loop structure.

**Conclusion:**

The binding of TLS and pncRNA-D was affected by the presence of GGUG and GGU sequences, and the C terminal domains of TLS function in the interaction with pncRNA-D.

**Electronic supplementary material:**

The online version of this article (doi:10.1186/s13578-016-0068-8) contains supplementary material, which is available to authorized users.

## Background

Translocated in LipoSarcoma [TLS, also known as FUsed in Sarcoma (FUS)] is an RNA/DNA binding protein whose mutation causes amyotrophic lateral sclerosis (ALS). Mutations in TLS, especially those at the C terminus of TLS, disrupt TLS transportation to the nucleus, and result in ALS [1–3]. In previous study, we demonstrated that long noncoding RNAs (lncRNAs) transcribed from the 5′ upstream region of cyclin D1 (*CCND1*), and these lncRNA are expected to bind to TLS and inhibits the histone acetyltransferase activity of CBP/p300 at the *CCND1* promoter. This interaction subsequently inhibits the expression of *CCND1* [4]. During this mechanism, binding between the lncRNA, promoter-associated noncoding RNA (pncRNA), and TLS changes the structure of TLS and allows it to interact with CBP/p300. Therefore, understanding the binding mechanism between TLS and pncRNA is an important issue in regulation of gene expression programs in eukaryotic cells.

Recent studies revealed that lncRNAs are mainly involved in gene silencing, but their mechanisms are not fully understood, especially for the binding between the lncRNA and the RNA binding proteins. Various lncRNAs have been reported to bind to RNA binding proteins [5]. One of the well-described lncRNA, Xist, binds to RNA binding protein SHARP to inhibit the expression of target genes [6]. Several reports revealed that TLS could also interact with thousands of RNAs including lncRNAs [7, 8].

TLS consists of SYGQ-rich domain, RNA recognition motif (RRM), zinc finger domain, and three RGG repeat domains. TLS binds to numerous number of RNAs, which are involved in cell cycle, RNA splicing, cellular response to stress and DNA repair, and so on [9]. RRM domain is usually expected to function in the binding between RNA, but whether RRM domain of TLS functions in the binding with RNAs is controversial [10–13]. According to our binding assay, pncRNA showed a strong binding to the C terminal domains of TLS but not to the N-terminal and RRM domains. Therefore, we examined the precise binding between lncRNA and the C terminal domains (RGG2–zinc finger–RGG3 domains and RGG3 domain) of TLS in this study.

There are several TLS recognition motifs reported by different groups, such as GGUG, GGU, GUGG, and CGCGC-motifs in RNAs [14–16], but its binding tends to be remarkably flexible. Wang et al. reported that TLS bind to RNA in the length dependent manner, and the mutation in the known binding motifs did not show a dramatic decrease in the binding [13], supporting the low specificity of the RNA binding of TLS.

In this study, we focused on one of the lncRNAs transcribed from the promoter region of *CCND1*, pncRNA-D, since it showed the highest expression level among the pncRNAs. We determined the full-length of pncRNA-D, and examined in which sequence TLS binds to by dividing pncRNA-D into seven fragments. We demonstrated that TLS strongly binds to 5′ and the 3′ ends of pncRNA-D, but could bind to fragments which do not contain any of the known binding motifs. Furthermore, we showed that the RGG2–zinc finger–RGG3 domains at the C terminal of TLS function in the interaction with pncRNA-D. In addition, the mutation in GGUG or GGU sequences dramatically decreased the binding between pncRNA-D and TLS.

## Results

### TLS bound strongly to the 5′ and the 3′ end of pncRNA-D

**Fig. 1:**
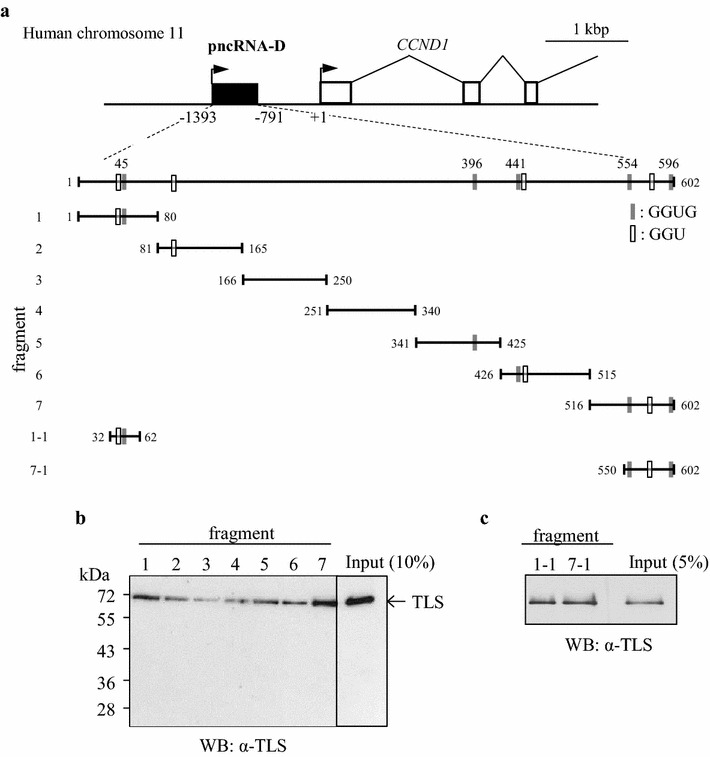
The binding between full-length TLS and fragmented pncRNA-Ds. **a** The position of pncRNA-D and *CCND1*. The fragmented pncRNA-Ds are shown at the *bottom*. *Black* and *white boxes* indicate GGUG and GGU sequence, respectively. Since fragment 3 and 4 did not contain any GGUG or GGU motifs, we considered them as a negative control. **b** and **c** Western blot analysis were conducted with HeLa nuclear extract (NE). Seven fragmented pncRNA-Ds (**b**) and shortened fragment 1 and 7 (**c**) were incubated with HeLa NE, and the affinity between TLS and each fragment was examined by RNA pull down assay. Five and ten percent of the protein used for RNA pull down assays were loaded as input *N* = 5

Among the lncRNAs expressed from the promoter region of *CCND1*, pncRNA-D showed the highest expression level in the previous study [4]. Therefore, we performed 5′ and 3′ RACE to determine the full-length of pncRNA-D. As a result, we found that pncRNA-D was a polyadenylated lncRNA with the length of 602 nt with single exon (Fig. 1a and Additional file 1: Figure S1). Then we examined if there were any open reading frames (ORFs) by ORF finder (http://www.ncbi.nlm.nih.gov/gorf/gorf.html). We detected three ORFs with length of 10–50 amino acids, but could not find any known protein domains in any of the expected short peptides (data not shown).

Since TLS is expected to bind to RNA with GGUG, GGU, GUGG, and CGCGC-motifs [14–16], we searched for these motifs in the 602 nt pncRNA-D, and found five GGUGs and five GGUs (excluding GGU in GGUG motif) at the positions indicated in Fig. 1a, but there was no CGCGC sequence. Next, we examined the binding between TLS and fragmented pncRNA-Ds. We divided pncRNA-D into seven fragments, and generated biotinylated RNA oligonucleotides. The result of binding assay indicated that full-length TLS protein were likely to interact with all the fragments investigated (Fig. 1b), though it showed a slight difference in the intensity of the binding. TLS bound strongly to the 5′ and 3′ end fragments of pncRNA-D, where GGUG and GGU sequences exist, but we have to note that TLS also bound to fragments 3 and 4 which do not have GGUG or GGU sequences, although the binding was weaker compared to other fragments.

We next examined more precise binding between TLS and fragments 1 and 7, since they showed stable interaction with TLS. Fragments 1 and 7 were shortened to 31 nt (fragment 1–1) and 53 nt (fragment 7–1) around GGUG sequences, respectively, and the result of binding assay demonstrated fragments 1–1 and 7–1 could still bind to TLS (Fig. 1c). The binding between TLS and random RNA oligonucleotide was also examined, but we only detected marginal interaction compared to pncRNA-D fragment 1–1 (Additional file 1: Figure S2).

### The secondary structure of the fragment 1–1 determined by NMR

**Fig. 2:**
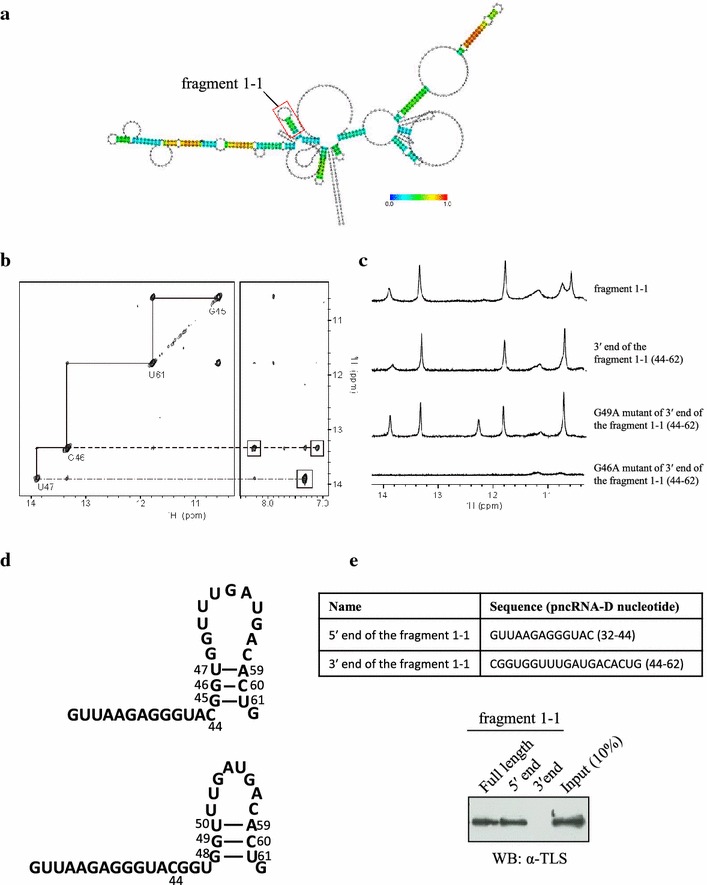
The binding between full-length TLS and the fragment 1–1. **a** Computational analysis predicting the secondary structure of pncRNA-D by CentroidFold (http://www.ncrna.org/centroidfold/). The position of the fragment 1–1 is shown in *red box*. **b** Imino-imino (*left*) and imino-amino/base (*right*) proton regions of a NOESY spectrum with a mixing time of 300 ms, the assignments of imino protons being indicated. Cross peaks to two amino protons and H2 are *boxed*, respectively. **c** 1D imino proton spectra of the fragment 1–1, the 3′ end of the fragment 1–1, G49A mutant of the 3′ end of the fragment 1–1 and G46A mutant of the 3′ end of the fragment 1–1, respectively, *from top to bottom*. **d** The two possible secondary structures of the fragment 1–1, the *upper one* being concluded as a right one. **e** Western blot analysis were performed to detect the binding between 5′ and 3′ ends of the fragment 1–1. Ten percent of the protein used for RNA pull down assay was loaded as input *N* = 5

We were interested in the fragment 1–1 (pncRNA-D nucleotides 32–62), since it showed the strong binding with TLS, and the in silico analysis implicated that the fragment 1–1 form stem loop structure (Fig. 2a) To determine the secondary structure of the fragment 1–1, NMR analysis was performed. Figure 2b shows the imino–imino and imino–amino/base proton regions of a NOESY spectrum of the fragment 1–1. The observation of a strong cross peak between two resonances at 10.6 ppm and 11.8 ppm (Fig. 2b, left) indicates the presence of a G:U base pair. The proton resonance at 13.3 ppm gave strong cross peaks to two amino protons (Fig. 2b, right), which indicates that the 13.3 ppm resonance is an imino proton one of G involved in a G:C base pair. The proton resonance at 13.9 ppm gave a strong cross peak to AH2 (Fig. 2b, right), which indicates that the 13.9 ppm resonance is an imino proton one of U involved in an A:U base pair. A cross peak between 11.8 and 13.3 ppm resonances and one between 13.3 and 13.9 ppm resonances indicates the presence of consecutive G:U, G:C and A:U base pairs for the fragment 1–1 [17, 18].

Then, the fragment 1–1 was divided into two fragments, the 5′ end (pncRNA-D nucleotides 32–44) and the 3′ end of the fragment 1–1 (pncRNA-D nucleotides 44–62, Fig. 2e top table). The imino proton spectrum of the 3′ end of the fragment 1–1 turned out to be almost identical to that of the fragment 1–1 (Fig. 2c), which indicates that the consecutive three base pairs found for the fragment 1–1 are formed in a 3′-region of the fragment 1–1 and that basically no base pair is formed in a 5′-region of the fragment 1–1. Figure 2d shows two possible secondary structures of the fragment 1–1 that involve the consecutive three base pairs. The imino proton spectrum of the G49A mutant of the 3′ end of the fragment 1–1 is similar to that of the 3′ end of the fragment 1–1, while almost no imino proton resonance is observed for the G46A mutant (Fig. 2c). This revealed that G49 is not critical for the formation of the secondary structure, while G46 is indispensable for the formation of the secondary structure. Thus, it is concluded that the fragment 1–1 formed the secondary structure shown in the upper of Fig. 2d.

We were interested in which end of the fragment 1–1 interacts with TLS, and performed the binding assay. Unexpectedly, TLS bound to the 5′ end of the fragment 1–1 and not to the 3′ end, which formed the stem loop structure (Fig. 2e, bottom). Taken together, TLS is likely to recognize the sequence of RNA rather than the secondary structure when binding the fragment 1–1.

### The C terminal zinc finger domain enhanced the specificity of the binding between TLS and pncRNA-D

**Fig. 3:**
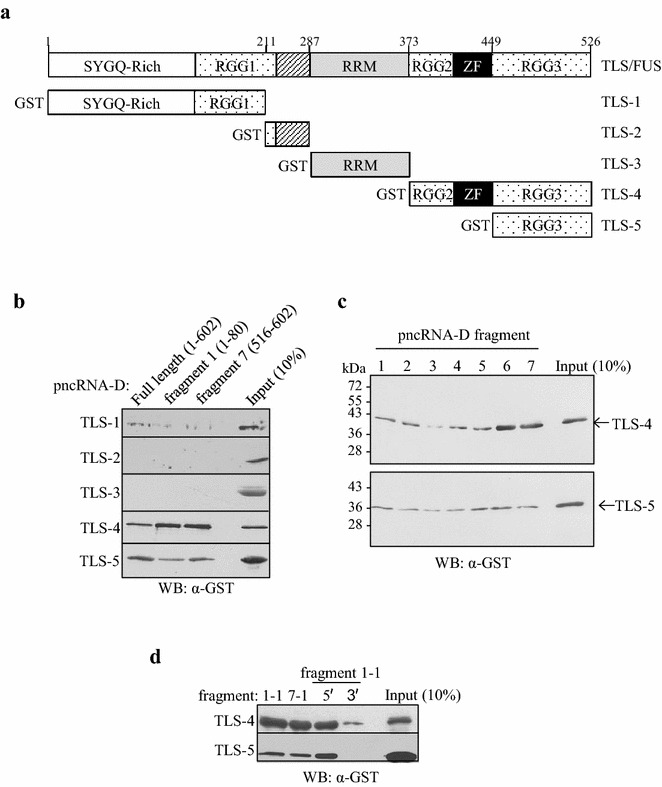
The binding between truncated TLS and pncRNA-D. **a** The domain structure of TLS. GST tag was attached to the 5′ end of the truncated TLS-1 to -5. *RGG* RGG repeat domain, *RRM* RNA recognition motif, *ZF* zinc finger domain. **b** The interaction between TLS-1 and -5 and pncRNA-D with different length was examined by RNA pull down assay. *N* = 3. **c** Binding of TLS-4 (*top*) and -5 (*bottom*) with fragmented pncRNA-D were examined by RNA pull down assay followed by western blot analysis. Purified TLS-4 and TLS-5 were incubated with pncRNA-D fragments, and the signals were detected with anti-GST antibody. *N* = 5. **d** The binding between the fragments 1–1, 7–1, the 5′ end and the 3′ end of fragment 1–1 with TLS-4 or TLS-5 were examined by RNA pull down assay as in (**b**) and (**c**). *N* = 4. In all the experiments, 10 % of the protein used for RNA pull down assay was loaded as input

Next we investigated the binding of truncated TLS fragments. We generated the GST-tagged TLS-1 to -5 as in Fig. 3a, and performed RNA pull down assay with full length pncRNA-D (602 nt), and pncRNA-D fragment 1 and 7, which showed an effective binding with full-length TLS (Fig. 1b). As a result, TLS-4 (TLS amino acids 373–526, containing RGG2, zinc finger, and RGG3 domains) and -5 (TLS amino acids 449–526, containing RGG3 domain) were able to bind to the RNAs examined. On the other hand, we observed merely slight binding of TLS-1 to the RNA oligonucleotides, and did not detect any significant binding of TLS- 2 or -3 (Fig. 3b).

We then investigated the binding of TLS-4 and -5 with RNAs examined in Fig. 1b. TLS-4 bound firmly to the 5′ end (fragment 1) and the 3′ end (fragment 7) of pncRNA-D, but slightly bound to fragments 3, 4 or 5 (Fig. 3c, top). TLS-5 was able to bind to all the RNA fragments, but its specificity decreased compared to full-length TLS and TLS-4 (Fig. 3c, bottom). We further examined the binding of TLS-4 and -5 to short pncRNA-D sequences (Fig. 3d). Both TLS-4 and -5 could bind specifically to the fragment 1–1, 7–1, and the 5′ end of the fragment 1–1, but bound slightly to the 3′ end of the fragment 1–1. These data suggest that TLS binds to pncRNA-D mainly through RGG domains, and zinc finger domain increased the specificity of the binding.

### The titration experiments with NMR revealed that TLS-5 interacts with a 5′-region of the fragment 1–1 more intensively than a 3′-region

**Fig. 4:**
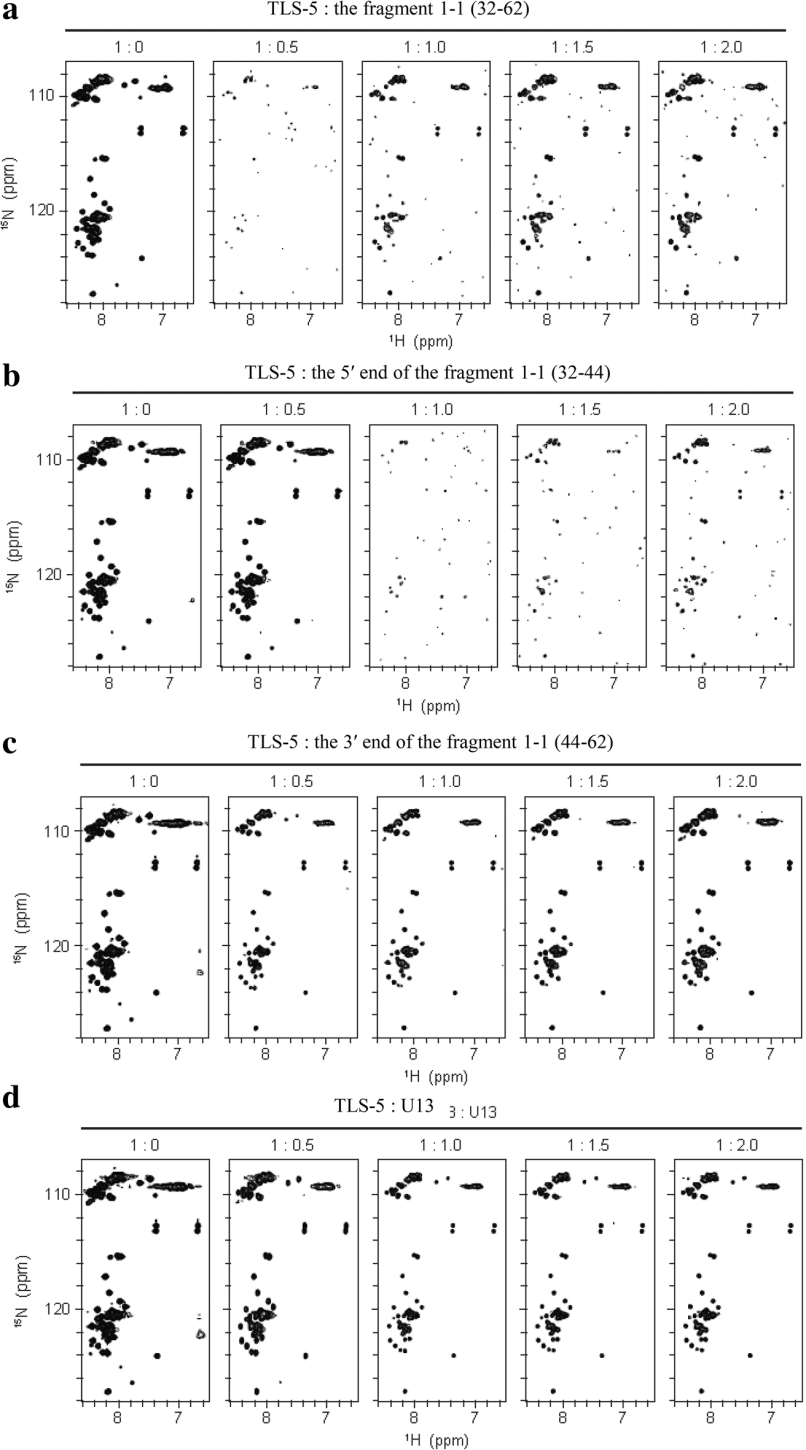
NMR titration experiments for TLS-5 with the fragment 1–1, the 5′ end of the fragment 1–1, the 3′ end of the fragment 1–1 and U13. **a** and **b** ^1^H–^15^N HSQC spectra of TLS-5 with either the fragment 1–1 (**a**), the 5′ end of the fragment 1–1 (**b**), the 3′ end of the fragment 1–1 (**c**) or U13 (**d**), with the molar ratios of 1:0, 1:0.5, 1:1.0, 1:1.5 and 1:2.0, respectively

Since the TLS showed a higher affinity to the 5′ end of the fragment 1–1 compared to the 3′ end of the fragment 1–1, which formed the stem loop structure, we further examined their binding with NMR analysis. In a course of the gradual addition of the fragment 1–1 to ^15^N-labeled TLS-5, ^1^H–^15^N HSQC correlation peaks of TLS-5 almost disappeared due to line broadening of each correlation peak at the 1:0.5 molar ratio, then, the correlation peaks re-appeared when the fragment 1–1 was further added (Fig. 4a). This kind of spectral change is typically observed when protein (TLS-5 in this case) in free and protein in complex with RNA (the fragment 1–1 in this case) undergoes the intermediate exchange regime in an NMR chemical shift time scale. Thus, the interaction of TLS-5 with the fragment 1–1 is clearly detected by NMR. The similar spectral change was observed when the 5′ end of the fragment 1–1 was gradually added (Fig. 4b). In this case, however, the disappearance occurred at the 1:1 molar ratio. The line broadening due to the intermediate exchange regime is known to be most remarkable when a half amount of protein is in a free form and another half amount of protein is in a complex form. In the case of the fragment 1–1, this situation was satisfied when the molar ratio was 1:0.5, while in the case of the 5′ end of the fragment 1–1, further addition of the 5′ end of the fragment 1–1 (the molar ratio of 1:1) was needed to reach this situation. This indicates that the 5′ end of the fragment 1–1 binds to TLS-5 less strongly than the fragment 1–1.

In a course of the gradual addition of the 3′ end of the fragment 1–1, in contrast, disappearance of correlation peaks did not occur, but gradual change of the positions of correlation peaks was observed (Fig. 4c). This kind of spectral change is typically observed when protein (TLS-5 in this case) in free and protein in complex with RNA (the 3′ end of the fragment 1–1 in this case) undergoes the fast exchange regime in an NMR chemical shift time scale. Usually, the affinity of the complex is lower for the fast exchange regime than for the intermediate exchange regime. Thus, it is suggested that the 3′ end of the fragment 1–1 binds to TLS-5 less strongly than the 5′ end of the fragment 1–1. The spectral change in a course of the gradual addition of U13 was similar to that of the 3′ end of the fragment 1–1 (Fig. 4d).

The dissociation constant of the TLS-5: the 3′ end of the fragment 1–1 complex was determined on the basis of the curve fitting on the gradual change of the positions of correlation peaks in a course of the gradual addition of the 3′ end of the fragment 1–1 (Additional file 1: Figure S3). The dissociation constant of 3.5 ± 1.9 × 10^−6^ M was obtained. The dissociation constant of the TLS-5: U13 complex was estimated in the same way to be roughly 2 × 10^−5^ M, although a precise value was difficult to be deduced due to weaker affinity. In a case of intermediate exchange regime, the dissociation constant cannot be obtained by curve fitting. As mentioned above, however, the affinity of the complex is usually lower for the fast exchange regime than for the intermediate exchange regime, probably at least by ca. 10 times. Thus, the dissociation constant of the TLS-5: the 5′ end of the fragment 1–1 complex was supposed to be approximately 3.5 × 10^−7^ M or even smaller. Finally, the dissociation constant of the TLS-5: the fragment 1–1 complex was supposed to be smaller than around 3.5 × 10^−7^ M. In summary, TLS-5 interacts with the 5′-region of the fragment 1–1 more firmly than a 3′-region.

### The mutation of GGUG and GGU sequences dramatically decreased the binding

**Fig. 5:**
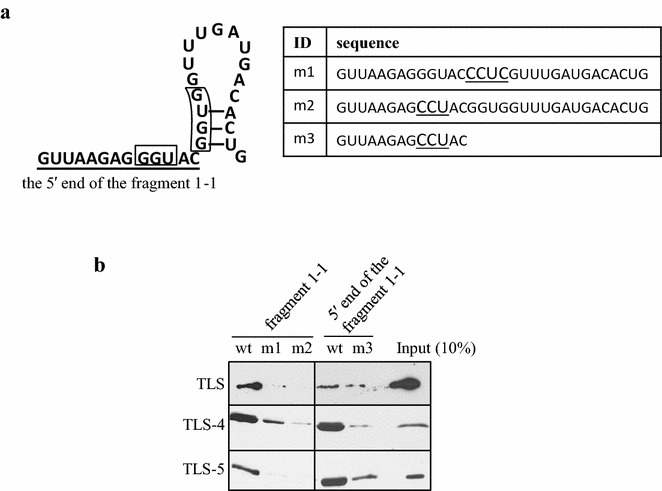
The effect of the mutation (GGUG to CCUC; GGU to CCU) on the binding of the fragment 1–1 and TLS. **a** The position of GGU and GGUG where mutation was induced in the fragment 1–1 is shown in *black box* (*left*), and the RNA sequences are listed in the table (*right*). Sequence of the 5′ end of the fragment 1–1 is *underlined*. **b** The binding between mutated fragments and full-length TLS, TLS-4, and TLS-5 was examined by RNA pull down assay *N* = 3. Ten percent of the protein used for RNA pull down assay was loaded as input

Finally, we examined the effect of the mutation of GGUG and GGU into CCUC and CCU, respectively, in the short RNA sequences on the TLS binding. The fragment 1–1 had GGU and GGUG sequence at the positions indicated in Fig. 5a, left. Therefore, we generated two mutated fragments in the fragment 1–1 (m1 and m2), and one for the 5′ end of the fragment 1–1 (m3) as listed in the table (Fig. 5a, right). As a result, each mutation dramatically reduced the binding of full-length TLS, TLS-4, and TLS-5 (Fig. 5b). Interestingly, the mutated fragments had a different effect in the binding. The m2 mutation (GGU to CCU at the 5′ end) decreased the interaction more effectively than the m1 mutation (GGUG to CCUC at the 3′ end) of the fragment 1–1. This data supports the fact that TLS preferentially binds to the 5′ end of the fragment 1–1 (Figs. 2e and 3d).

## Discussion/Conclusion

Significant percentages of the human genome have been shown to be transcribed into RNAs, while most of them are supposed to be lncRNAs. Therefore, it is increasing impact on assigning functions to these lncRNA. However, relatively small numbers of reports have been showing biological functions of lncRNAs because of lacking full-length sequence and structural data of lncRNAs. In this study, we have determined the exact RNA sequence of pncRNA-D and deduced specific secondary structures with NMR analysis. These data present supportive information which boosts whole area of the lncRNA investigations.

There are several experimental techniques employed to detect the protein–RNA interactions, such as electrophoretic mobility shift assay (EMSA), FISH assay, and RNA pull-down assay [13, 19, 20]. In the previous study, we employed EMSA [4], because it is commonly used technology to detect RNA binding to proteins. However, we encountered some difficulties, for instances, instability of detection of signals, and handling of relatively higher exposure to radioactivity of ^32^P-probes. Therefore, we have developed the RNA pull down assay using biotinylated RNA oligonucleotides with TLS. The assay presents more reproducible data than one with EMSA. Another option for detection of RNA in cells is FISH assay. FISH assay is not applicable to our experiments since the expression level of pncRNA-D was under the detection level with it.

TLS preferentially bound the 5′ and 3′ regions (fragments 1, 2 and 6, 7) of the seven fragments over the full-length pncRNA-Ds, although certain levels of binding were detected in the middle fragments (fragments 3, 4, and 5) (Figs. 1b and 3b). The both edges of the pncRNA-D have the GGUG and GGU motifs and the middle fragments does not possess them, suggesting that these motifs could confer the binding preference for TLS. Actually, the mutational experiments using the RNA oligonucleotides of pncRNA-D (nucleotides 32–62: the fragment 1–1) with the GGUG and GGU motifs confirm that they have crucial roles in the binding to TLS in biochemical environments (Fig. 5b). *In silico* analysis predicted that the 3′ end of the fragment 1–1 forms the stem loop structure. The NMR analysis confirmed that the 3′ end of the fragment 1–1 forms the stem loop structure (Fig. 2) and the 5′ end does not form any stable structure indicating the 5′ end as natively unfolded status. It has been reported that TLS preferentially bind to the stem loop structure [7, 14]. Contrast to previous reports, our experiments demonstrated that TLS bound to the 5′ end of the fragment 1–1, bearing natively unfolded status instead of the 3′ end, which forms the stem loop structure.

Comparing the binding of RGG3 domain of TLS (TLS-5) to the fragment 1–1 and just the 5′ end of the fragment 1–1 with the NMR analysis, the whole fragment 1–1 (pncRNA-D nucleotide 32–62) turned out to have more solid binding than the 5′ end of the fragment 1–1 (pncRNA-D nucleotide 32–44) (Fig. 4). On the other hand, the RNA pull down assay (Fig. 3d) showed that TLS-5 bound more firmly to the 5′ end of the fragment 1–1 than the 3′ end. In addition, presence of the 3′ end of the fragment 1–1 (pncRNA-D nucleotide 44–62) reduced the interaction between TLS-5 and the fragment 1–1 (Fig. 5b, comparing the binding between m2 and m3). This discrepancy of these two results might be caused from the difference in experimental conditions between the NMR analysis and the RNA pull down assay. However, our preliminary RNA pull down experiments incubating the nuclear extract of HeLa cells with both 5′ and the 3′ ends of the fragment 1–1 indicated that both edges captured certain numbers of proteins (data not shown). These data indicate that the 3′ end of the fragment 1–1 might play a regulatory role on the interaction between the RNA and TLS.

The interaction of full-length TLS and TLS-4 with seven fragments of pncRNA-D showed a similar profile (Figs. 1b and 3b). On the other hand, the binding of TLS-5 to these RNA fragments was more labile and the specificity tends to be lower compared to the full-length TLS and TLS-4. These data demonstrate that the RGG2–zinc finger–RGG3 domain (TLS-4) is mainly involved in the binding with pncRNA-D, and the zinc finger domain enhances the specificity and the intensity of the binding. Indeed, the zinc finger domain could enhance the protein–RNA interaction [21, 22].

TLS has been found to accommodate more than 30,000 RNA species (data not shown). Our binding experiments in this study indicated that some RNA molecules have high specificity and others have low specificity, inspiring that TLS binds to RNA in a different molecular mechanism. In this study we identified some RNA sequences bound to TLS with high specificity (for example, at the fragments 1 and 7), other RNA sequences bound to TLS with low specificity (at the fragments 3 and 4), inspiring that TLS binds to these two categories of RNA fragments through distinctive surface(s) of the molecule. For another example, the Thomas Cech group reported that TLS binds to RNAs without any identified recognition motifs (such as GGUG), and mutations in those motifs slightly decrease the related bindings [13]. These are a category of the RNA binding with low specificity. Contrarily, our experiments in this study have shown a distinctive category of the RNA binding, the highly specific binding of TLS to pncRNA-D through characteristic motifs like GGUG and GGU (Fig. 5). Taken together, we present that distinct specificities of the RNA binding of TLS functions in cells, suggesting that they should play each specific role in supporting biological events each in development and homeostasis programs of eukaryotes.

## Methods

### Cell culture and nuclear extract preparation

Hela cells were cultured as previously described [23]. For nuclear extract preparation, HeLa cells were cultured in 15 cm dishes. HeLa cells were washed with PBS and collected in 5 ml phosphate buffered saline (PBS), and centrifuged at 800×*g* for 5 min. Supernatant was discarded and the cells were resuspended in 2.5 ml of Hypotonic buffer (10 mM HEPES, 1.5 mM MgCl_2_, 10 mM KCL, 0.5 mM DTT, 0.2 mM PMSF), and centrifuged at 1100×*g* rpm for 1 min. After removing all the supernatant, the cells were resuspended in 10 ml Hypotonic buffer, and kept on ice for 10 min. The cells were homogenized with digital homogenizer (1500 rpm, 10 min with gentle up and down), and the samples were centrifuged at 1100×*g* for 20 min (precipitation is nuclei). The nuclei were resuspended in High salt buffer (20 mM HEPES, 1.5 mM MgCl_2_, 0.6 M NaCl, 0.2 mM EDTA, 25 % Glycerol, 0.5 mM DTT, 0.2 mM PMSF), and rotated for 30 min in 4 °C. The sample was centrifuged at 20,000×*g* for 30 min, and the supernatant was collected and dialyzed in dialysis buffer (20 mM HEPES, 0.1 M NaCl, 0.2 mM EDTA, 20 % Glycerol, 0.5 mM DTT, 0.2 mM PMSF) for 18 h.

### 5′ and 3′ RACE

5′ and 3′ RACE were performed using 5′-Full RACE Core Set (TaKaRa) and 3′-Full RACE Core Set (TaKaRa) according to the manufacturer’s protocols. The primers used in 5′ RACE were 5′ end-phosphorylated RT Primer: P-GGACTGAATTCGTG, 1st PCR primers: S1:GTTTAATTGATAATTGTTCTG and A1:CGAGTGTACTGATCTGAT, 2nd PCR primers: S2:ATTATGCCGGCTCCTGCCAG and A2:CGATAGGTGTTCGTGGTTAC. Primers used in 3′ RACE were Reverse transcription primer; oligo(dT)-containing Adapter Primer: GGCCACGCGTCGACTAGTACTTTTTTTTTTTTTTTTT, 1st PCR primers: Gene-Specific Primer 1 (GSP 1): TTTTTCTATCAGTTTTCTTTGAGCTTTTAC, Abridged Universal Amplification Primer 1(AUAP 1): GGCCACGCGTCGACTAGTAC, 2nd PCR primers: GSP2: GAGGGTACGGTGGTTTGATGACACTGAAC, AUAP2: GGCCACGCGTCGACTAG, and 3^rd^ PCR primers: AUAP2 and GSP3: GCACCAAAGAGACAGAACCTGTAATTTTAAAAACTGTG.

### Overexpression of GST–TLS-4

GST–TLS-4 were overexpressed and purified as previously described [24]. Briefly, the ORF of TLS-4 was inserted into pGEX-KG vector, and transfected into Y1090. The expression of GST–TLS-4 were induced by IPTG, and the protein were purified with Glutathione beads. The purified GST–TLS-4 proteins were used for RNA binding assay.

### RNA pull down assay

Biotinylated RNAs (fragmented pncRNA-Ds, mutated the fragment 1–1 and the 5′ end of fragment 1–1) were purchased from Integrated DNA Technologies MBL. Dynabeads-M280 was washed with PBS/0.02 % tween 20, and resuspended with whole cell extract (WCE) buffer containing tRNA (1 μg/μl) and RNase Inhibitor (4 U/μl). 20 pmol RNA oligonucleotides were added to the beads, and incubated for 15 min at room temperature with rotation. After 15 min of rotation, 100 μl of HeLa cell nuclear extract (to detect full-length TLS) or 3 μg of GST–TLS-4 or -5 were added and incubated for 1 h at 4 °C with rotation. The beads were washed with 1 ml WCE buffer for four times, and resuspended in 2× sodium dodecyl sulfate (SDS) sample buffer (0.25 M Tris–HCl pH 6.8, 4 % SDS, 10 % 2-mercaptoethanol, 20 % glycerol). The samples were boiled at 100 °C for 3 min, and the dynabeads were removed by a magnetic stand. The supernatants were used for western blot analysis.

### Western blot analysis

Western blot analysis was performed as previously described [23]. Briefly, the membranes were incubated with anti-TLS (BD bioscience, 611385) or anti-GST antibody (Santa Cruz sc-459) for 1 h at room temperature. Then the membranes were washed with PBST for 5 min, four times, and incubated with anti-mouse HRP-conjugated IgG (Dako, P0161) or anti-rabbit HRP-conjugated HRP (Cell signaling, 70745). All the antibodies were diluted by 1 % skim milk to 1:2000. The signals were detected with SuperSignal West Pico substrate (Thermo Scientific).

### RNA

The fragment 1–1 [r(GUUAAGAGGGUACGGUGGUUUGAUGACACUG)], the 5′ end of the fragment 1–1 [r(GUUAAGAGGGUAC)], the 3′ end of the fragment 1–1 [r(CGGUGGUUUGAUGACACUG)], G46A mutant of the 3′ end of the fragment 1–1 [r(CGAUGGUUUGAUGACACUG)], G49A mutant of the 3′ end of the fragment 1–1 [r(CGGUGAUUUGAUGACACUG)] and U13 [r(UUUUUUUUUUUUU)] were synthesized, purified by HPLC and desalted by FASMAC Co., Ltd and Japan Bio Services Co., Ltd.

### Expression and purification of ^15^N-labelled TLS-5

The plasmid containing TLS-5 fused to GST was a generous gift from Dr. Takanori Oyoshi (Shizuoka University). The expression and purification of GST–TLS-5 basically followed the reported protocol [24] except for using minimal medium (M9) containing 1 g/1L ^15^N-ammonium chloride to obtain ^15^N-labeled protein [25]. To improve the purity, cation exchange chromatography was also used in purification. The purified protein was dialyzed against titration buffer, 10 mM sodium phosphate buffer (pH 6.5) containing 100 mM NaCl, 1 mM MgCl_2_ and 0.01 mM 2,2-dimethylsilapentane-5-sulfonic acid (DSS), and concentrated with Amicon Ultra (MWCO 3500, Millipore).

### NMR spectroscopy

For the analysis of the secondary structure, the fragment 1–1, the 5′ end of the fragment 1–1, the 3′ end of the fragment 1–1, G46A mutant of the 3′ end of the fragment 1–1, and G49A mutant of the 3′ end of the fragment 1–1 were dissolved, respectively, in ca. 10 mM potassium phosphate buffer (pH 6.2) containing 10 mM KCl and 0.01 mM DSS. For the titration experiments, the fragment 1–1, the 5′ end of the fragment 1–1, the 3′ end of the fragment 1–1 and U13 were dissolved, respectively, in the titration buffer described above. Each RNA was added step by step to a ^15^N-labeled TLS-5 solution with molar ratios of 1:0, 1:0.5, 1:1.0, 1:1.5 and 1:2.0. NMR spectra were recorded with a Bruker AVANCE III HD 600 spectrometers equipped with a cryogenic probe with a Z-gradient at 5 °C. Chemical shift was calibrated with a DSS resonance. NMR data were processed and analyzed using TopSpin/XWIN-NMR (Bruker), NMRPipe [26] and Sparky (http://www.cgl.ucsf.edu/home/sparky/).

## Additional file

10.1186/s13578-016-0068-8 **Figure S1**. The full length sequence of pncRNA-D. **Figure S2**. The binding between full-length TLS, TLS-4 and 5 with Random 30 nt RNA. **Figure S3**. A representative fitted curve for the titration experiment by NMR.

## Abbreviation

ALS: amyotrophic lateral sclerosis
CCND1: cyclin D1
EMSA: electrophoretic mobility shift assay
FISH: fluorescence in situ hybridization
FUS: FUsed in Sarcoma
HRP: horseradish peroxidase
lncRNA: long noncoding RNA
NMR: nuclear magnetic resonance
PBS: phosphate buffered saline
pncRNA: promoter-associated ncRNA
RRM: RNA recognition motif
SDS: sodium dodecyl sulfate
TLS: Translocated in LipoSarcoma
WCE: whole cell extract
