# Supplementary material for: The binding specificity of Translocated in LipoSarcoma/FUsed in Sarcoma with lncRNA transcribed from the promoter region of cyclin D1
Source: Cell Biosci. 2016 Jan 25;6:4. doi: 10.1186/s13578-016-0068-8 (PMC4727290; doi:10.1186/s13578-016-0068-8)
Supplement: Supplementary file 1 — 10.1186/s13578-016-0068-8 Figure S1. The full length sequence of pncRNA-D. Figure S2. The binding between full-length TLS, TLS-4 and 5 with Random 30 nt RNA. Figure S3. A representative fitted curve for the titration experiment by NMR. [file 13578_2016_68_MOESM1_ESM.pdf]

UUUUUCUAUCAGUUUUCUUUGAGCUUUUACUGUUAAGAGGGUACGGUGGUUUGAUG
ACACUGAACUAUAUUCAAAAGGAAGUAAAUGAACAGUUUUCUUAAUUUGGGGCAGG
UACUGUAAAAAUAAAAACAAAAGUUAAGACAGUAAAAUGUCCUUUUAUUUUUUAAU
GCACCAAAGAGACAGAACCUGUAAUUUUAAAAACUGUGUAUUUUAAUUUACAUCUG
CUUAAGUUUGCGAUAAUAUUGGGGACCCUCUCAUGUAACCACGAACACCUAUCGAUU
UUGCUAAAAAUCAGAUCAGUACACUCGUUUGUUUAAUUGAUAAUUGUUCUGAAUUA
UGCCGGCUCCUGCCAGCCCCCUCACGCUCACGAAUUCAGUCCCAGGGCAAAUUCUAA
AGGUGAAGGACGUCUACACCCCCAACAAAACCAAUUAGGAACCUUCGGUGGUCUUG
UCCCAGGCAGAGGGGACUAAUAUUUCCAGCAAUUUAAUUUCUUUUUUAAUUAAAAA
AAAUGAGUCAGAAUGGAGAUCACUGUUUCUCAGCUUUCCAUUCAGAGGUGUGUUUC
UCCCGGUUAAAUUGCCGGCACGGGAAGGGAGGGGGUGCA

Supplementary Figure S1. The full length sequence of pncRNA-D
The sequence of pncRNA-D determined by RACE assay is shown. Positions of GGUG is underlined.

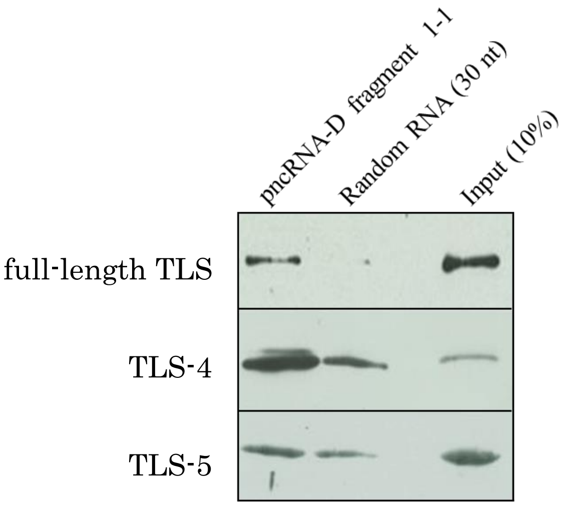

Supplementary Figure S2. The binding between full-length TLS, TLS-4 and 5 with Random 30 nt RNA.

RNA pull down assay was conducted with biotinylated RNAs (pncRNA-D fragment 1-1 and Random 30 nt RNA: UAG GCA AAG GAC UCA CCU UUG AAU UGC GUC) as described in material and methods. The random 30 nt RNA do not contain GGUG or GGU sequences. *N*= 3.

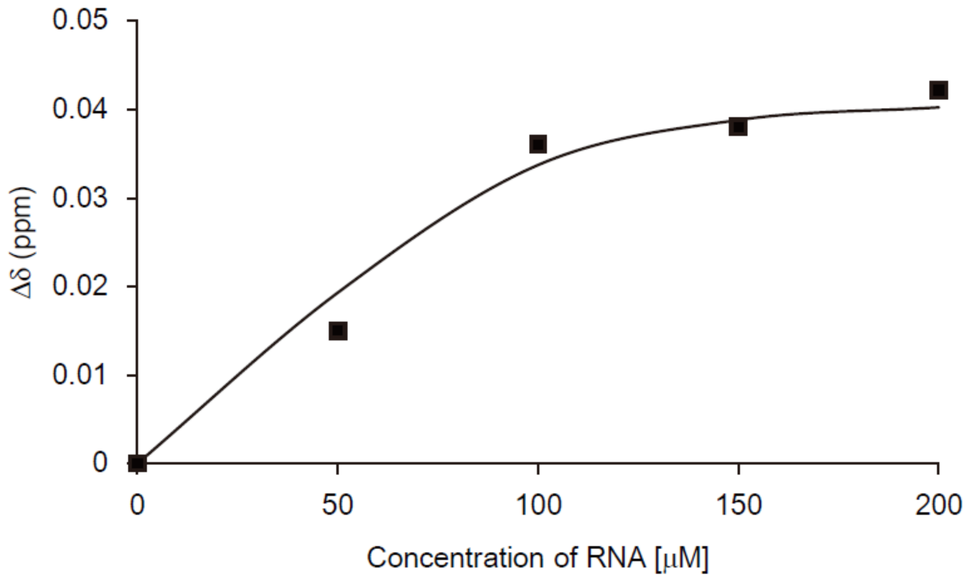

Supplementary Figure S3. A representative fitted curve for the titration experiment by NMR. Experimentally obtained Δδ values for one correlation peak in the course of the addition of 3' end of fragment 1-1 to TLS-5 are indicated by squares and a fitted curve is shown by solid line.

Material and Methods

Determination of the dissociation constant (K_D) of the TLS-5:3' end of fragment 1-1 complex on the basis of the titration experiment by NMR

Six correlation peaks of ^1H-^{15}N HSQC spectrum of TLS-5 whose positions largely changed in the course of the addition of the 3' end of the fragment 1-1 were used to determine the dissociation constant (K_D). Chemical shift difference, Δδ, was defined as $\Delta\delta = [(\Delta\delta_H)^2+(\Delta\delta_N/6.5)^2]^{1/2}$, where $\Delta\delta_H$ and $\Delta\delta_N$ are the chemical shift differences for H^N and ^{15}N, respectively. Δδ at each molar ratio was fitted by the following equation [S1] using Microsoft Excel's Solver:

$$\Delta\delta = \Delta_{max}\frac{(K_D+[L]_0+[P]_0)-\sqrt{(K_D+[L]_0+[P]_0)^2-(4[P]_0[L]_0)}}{2[P]_0}$$

where Δ_{max} is the maximum chemical shift difference at saturation, $[P]_0$ and $[L]_0$ and are the total concentrations of TLS-5 and the 3' end of the fragment 1-1, respectively. The average and standard deviation of six K_D values obtained from each correlation peak were calculated. Then, K_D was determined to be $3.5 \pm 1.9 \times 10^{-6}$ M.

Reference

S1 Fielding L: NMR methods for the determination of protein-ligand dissociation constants. *Prog Nucl Magn Reson Spectrosc* 2007, 51:219-242.
